# Differential engulfment of *Staphylococcus aureus* and *Pseudomonas aeruginosa* by monocyte-derived macrophages is associated with altered phagocyte biochemistry and morphology

**DOI:** 10.17179/excli2020-2766

**Published:** 2020-09-30

**Authors:** Mohamed El Mohtadi, Lisa Pilkington, Christopher M. Liauw, Jason J. Ashworth, Nina Dempsey-Hibbert, Amina Belboul, Kathryn A. Whitehead

**Affiliations:** 1 Department of Biology, Edge Hill University, Ormskirk, Lancashire, L39 4QP, UK; 2 School of Chemical Sciences, University of Auckland, Auckland 1010, New Zealand; 3 Microbiology at Interfaces, Faculty of Science and Engineering, Manchester Metropolitan University, Manchester M1 5GD, UK; 4 Faculty of Science and Engineering, Manchester Metropolitan University, Manchester M1 5GD, UK

**Keywords:** macrophages, bacteria, phagocytosis, FTIR, cell membrane, morphology

## Abstract

Knowledge of changes in macrophages following bacterial engulfment is limited. U937-derived macrophages were incubated with *Staphylococcus aureus* or *Pseudomonas aeruginosa*. Morphological and biochemical changes in macrophages following host-pathogen interactions were visualized using Scanning Electron Microscopy (SEM) and Fourier-Transform Infrared Spectroscopy (FTIR) respectively. Principal Component Analysis (PCA) was used to assess the variability in the FTIR spectra. Following host-pathogen interactions, survival of *S. aureus *was significantly lower than *P. aeruginosa* (P<0.05) and cellular morphology of macrophages was different after incubation with *S. aureus* compared to *P. aeruginosa. *Following incubation with *S. aureus* macrophages were more globular and amorphous in shape whereas long linear pseudopodia were observed following incubation with *P. aeruginosa. *Distinct FTIR spectra were identified in macrophages post interaction with the different bacteria and PCA analysis demonstrated distinct biochemical differences in the phagocytes following engulfment of the bacteria, with > 99 % of variability in the FTIR spectra explained by the first two principal components. These findings demonstrated that there were clear morphological and biochemical changes in macrophages following engulfment of two different bacterial types suggesting that the biochemical components of the bacterial cell wall influenced the biochemical characteristics and hence the morphology of macrophages in distinct ways.

## Introduction

Macrophages are host immune cells characterized by the ability to engulf and digest bacteria, damaged cells and foreign substances via a process known as phagocytosis (Duque and Descoteaux, 2015[7]). Once a bacterial cell gains entry to a human host, phagocytosis is fundamental to the immune response and the elimination of pathogens since it involves their entrapment by host cells (Desjardins et al., 2005[6]). Unsuccessful or imperfect clearance of bacteria are linked to several pathological conditions such as chronic inflammation, wound infections and some autoimmune diseases (Engelich et al., 2001[8]). Phagocytosis is a dynamic biological process involving rapid changes in host cell morphology in response to foreign invaders. This results in actin polymerization and re-arrangement of the actin cytoskeleton to form extensions of the plasma membrane called pseudopodia (Freeman and Grinstein, 2016[11]). The pseudopodia form a phagocytic cup that surrounds the bacterium and the microorganism is then internalized within a phagosome (Jilkine, 2009[18]). The membrane-bound phagosome subsequently fuses with a lysosome to form a phagolysosome (Freeman and Grinstein, 2016[11]; Garcia-Gomez et al., 2016[12]), and destruction of the engulfed bacterium is mediated by lysosomal enzymes and reactive oxygen species (ROS) within the phagolysosome (Pauwels et al., 2017[31]).

Recognition of bacteria by the body's immune system involves recognition of bacterial cell wall components, such as lipoproteins in Gram-positive bacteria or lipopolysaccharide (LPS) in Gram-negative bacteria (Triantafilou and Triantafilou, 2002[45]). *Pseudomonas aeruginosa* (Gram-negative) and *Staphylococcus aureus* (Gram-positive) are clinically significant pathogens, due to their propensity to cause infection and their resistance to conventional antibiotics. *P. aeruginosa* have two membrane bilayers; an asymmetric outer membrane, mainly composed of LPS in the outer bilayer and phospholipids in the inner bilayer, and the inner bilayer that is composed of phospholipids (Gyorfy et al., 2013[15]). The cell wall of *S.* *aureus* is composed of teichoic acids that are anionic glycopolymers cross-stitched to a thick layer of peptidoglycan (Xia et al., 2010[49]).

Although bacteria possess characteristics and mechanisms to evade innate host responses (Cossart and Sansonetti, 2004[5]), research to investigate whether macrophages undergo distinct morphological and biochemical changes in response to differing bacteria has received little attention to date.

FTIR spectroscopy is a biophysical method that can be used for characterizing the biochemical composition of cells, since small changes in the peptide-membrane interactions, lipid dynamics, membrane protein conformation dynamics or products of metabolic pathways, can lead to changes in the FTIR fingerprint (Movasaghi et al., 2008[28]). FTIR has been used for both mammalian and bacterial cell lines to determine the subtle alterations in both the conformation and concentration of macromolecules (Liu et al., 2003[23]). The FTIR spectra of intact cells reflect the vibrational or rotational motions of specific functional groups or bonds in biochemical components such as proteins, lipids, and carbohydrates within cell membranes (Naumann et al., 1996[29]). The use of such analytical techniques can provide new information on biochemical processes involved in the functioning of eukaryotic cells. Thus, the aim of this work was to use Scanning Electron Microscopy (SEM) and Fourier-Transform Infrared (FTIR) spectroscopy to determine the morphological and biochemical changes in macrophages during phagocytosis of two different bacteria, *S. aureus *and *P. aeruginosa.*

## Materials and Methods

### Preparation of RPMI-1640 complete medium

Roswell Park Memorial Institute (RPMI)-1640 (L-Glutamine, 25 mM HEPES) (Lonza, UK) supplemented with 10 % heat-inactivated fetal bovine serum (FBS) and 2 % penicillin-streptomycin (Lonza, UK) was prepared under aseptic conditions and referred to as RPMI-1640 complete medium throughout the study.

### Cell culture

U937 monocytes (Health Protection Agency Culture Collections, UK) were cultured under aseptic conditions at 37 °C and 5 % CO_2_ using RPMI-1640 complete medium. Monocytes were maintained at 0.5 x 10^6^ cells/ml by resuspension in fresh RPMI-1640 complete medium every other day. Sterile filtered 0.4 % trypan blue (Lonza, UK) dye was used to check cell viability according to published methodologies (Mir et al., 1991[27]; Sproston et al., 2018[42]), using a 1:1 ratio of cell suspension to trypan blue and counting the number of non-viable cells that take up the blue dye using a TC10 automated cell counter (Bio-Rad, USA). The viability of cells was above 80 % for all experiments.

### Differentiation of monocytes into macrophages in vitro

U937 monocytes at a concentration of 1 x 10^6^ cells/ml were differentiated into macrophage-like cells by incubating with 50 ng/mL phorbol 12-myristate 13-acetate (PMA) (Applichem, Germany) in RPMI complete medium for 24 hours at 37 °C and 5 % CO_2_. Cells were washed twice with complete medium and then rested for a further 48 hours in fresh PMA‐free complete medium to obtain resting macrophages (Sproston et al., 2018[42]; Rios de la Rosa et al., 2017[37]).

### Bacterial culture preparation

Methicillin-resistant *S. aureus* (MRSA) strain 11 (Bolton Hospital isolates, Manchester, UK) and *P. aeruginosa* strain PAO1 (Bolton Hospital isolates, Manchester, UK) were used throughout the work. When required for experimental assays, *S. aureus* and *P. aeruginosa* were cultured in nutrient broth (NB) (Oxoid, UK) overnight at 37 °C on an orbital shaker (New Brunswick Scientific, USA). The broth was centrifuged at 1750 *g* for 10 minutes and the bacterial cell pellets washed twice with saline prior to re-suspension at 2 x 10^5^ CFU/mL in antibiotic-free RPMI-1640 complete medium.

### Host-pathogen interactions assay

Monocyte-derived macrophages were prepared in a 24-well plate. Macrophages were stimulated with 1 µg/mL LPS and 100 ng/mL IFN-γ in antibiotic-free medium for 2 hours at 37 °C and 5 % CO_2_. The supernatant was removed from all wells before inoculating the macrophages with or without 1 x 10^4 ^CFU of *S. aureus* or *P. aeruginosa* in antibiotic-free RPMI-1640 complete medium. The plates were incubated for 3 hours at 37 °C and 5 % CO_2_ to enable host-pathogen interaction (phagocytosis) to occur. Following the 3-hour host-pathogen interaction period, the constituents of each well were collected and inoculated onto nutrient agar (NA) plates (Oxoid, UK) in duplicate and incubated at 37 °C overnight. The number of CFU/mL recovered on the agar plates were utilized to calculate the bacterial recovery in each sample following the period of host-pathogen interaction (n=6).

### Scanning electron microscopy of macrophages with S. aureus or P. aeruginosa

Sterile silicon wafers of 1 cm^2^ diameter were washed with DPBS (Sigma-Aldrich, UK) for 10 minutes before being placed in a 12-well plate. Adherent monocyte-derived macrophages (1 x 10^6^ viable cells) were seeded onto the silicon wafers and transferred into antibiotic-free RPMI-1640 complete medium. The supernatant was removed from all wells prior to incubating macrophages with 1 µg/mL LPS and 100 ng/mL IFN-γ in antibiotic-free medium for 2 hours at 37 °C and 5 % CO_2_. The supernatant was removed from all wells prior to incubation of macrophages with or without 1 x 10^5 ^CFU of *S. aureus* or *P. aeruginosa* in antibiotic-free RPMI-1640 complete medium, resulting in a 10:1 ratio of macrophages to bacteria in those wells inoculated with bacteria. The cultures were incubated for 3 hours at 37 °C and 5 % CO_2_ to allow host-pathogen interactions to occur. Following the 3-hour period of host-pathogen interface, samples were fixed using 2.5 % glutaraldehyde in DPBS (Sigma-Aldrich, UK) and incubated at 4 °C overnight. Wafers were washed twice in DPBS, then consecutively washed in 20 %, 40 %, 60 %, 80 % and finally in 100 % methanol/ethanol (Fisher Scientific, UK) for 30 minutes each, before being dried overnight in a vacuum-assisted desiccator (Sigma-Aldrich, UK). A Supra 40VP SEM (Zeiss, Germany) was used to capture images of bacterial internalization using SmartSEM software (Carl Zeiss Ltd, Germany) (n=3).

### Fourier Transform Infrared Spectroscopy (FTIR)

A FTIR microscope (Thermo-Winslet Continuum, UK) attached to a FTIR spectrophotometer (Thermo Nicolet Nexus, UK) bench was used to analyze samples (silicon wafers). The microscope aperture was set to 200 mm x 200 mm and fitted with a type A MCT detector. The FTIR spectra of macrophages incubated with or without bacteria were made up of 52 scans (Omnic 5.2 software, UK) (n=6).

### Principal Component Analysis (PCA)

PCA of the FTIR data (range: 400-4000 nm^-1^) was carried using R (version 3.2.2) and R Studio (version 0.99.486) (R Core Team, 2015[33]; RStudio Team, 2015[38]). The PCA analysis was performed using the prcomp function as part of the stats package, by singular value decomposition of the centred and scaled data matrix (R Core Team, 2015[33]). Results of this analysis were visualized using the factoextra package (version 1.0.5) (Kassambara and Mundt, 2016[20]).

Microsoft Excel and IBM SPSS Statistics (Version 25) were used to analyze all data. Parametric Student's t test was conducted to determine significance; P values <0.05 were considered as statistically significant.

## Results

The number of bacteria recovered from the host-pathogen interface assay (Table 1(Tab. 1)) demonstrated significantly (P<0.05) lower survival of *S. aureus *than *P. aeruginosa* survival (18.9 % and 24.1 %) following a 3-hour period of host-pathogen interactions.

**Figure 1:**
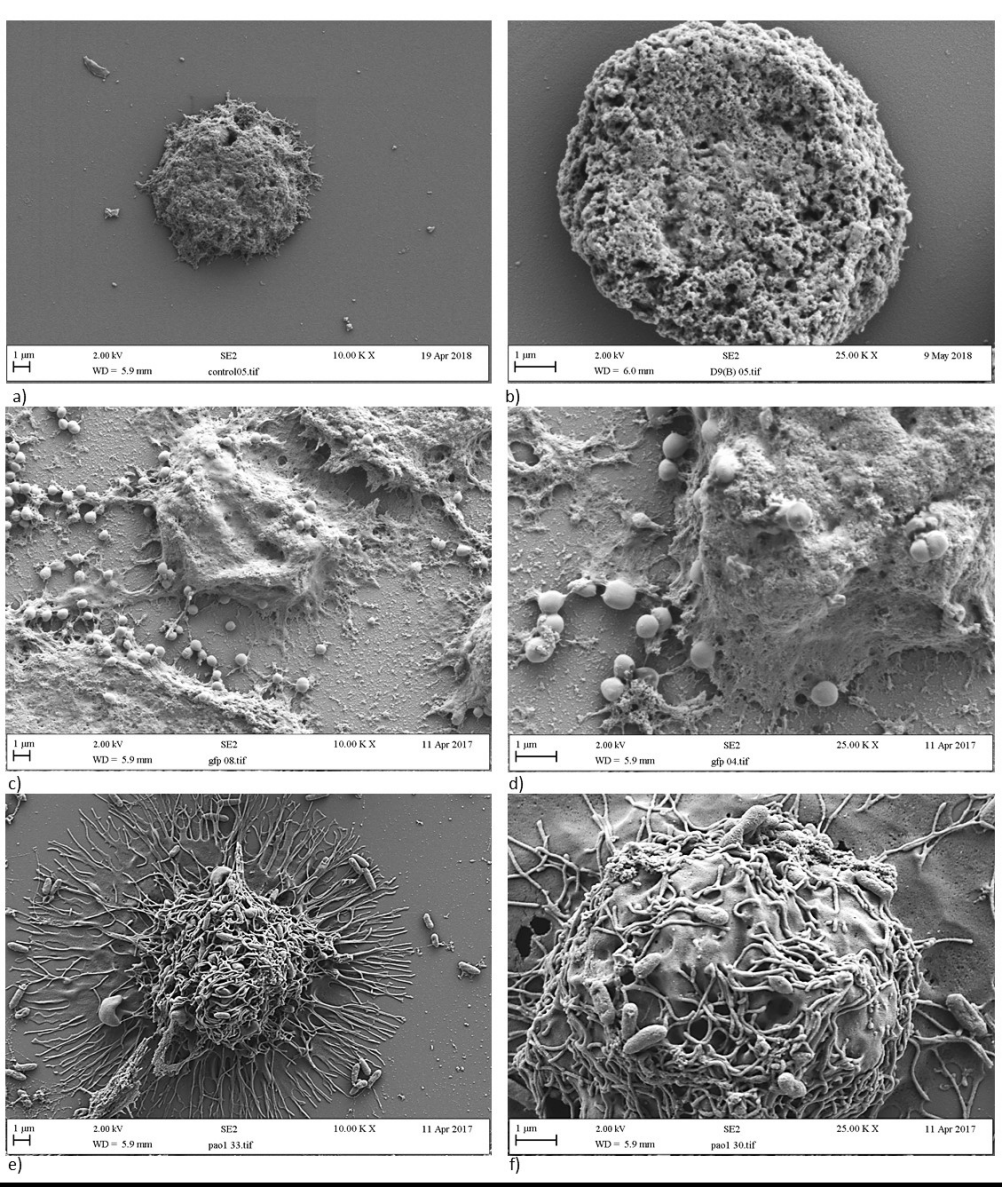
Morphology of a/b) resting macrophages, c/d) macrophages engulfing *S. aureus,* and e/f) macrophages engulfing *P. aeruginosa*. Panels a, c and e are images at x 10,000 magnification whereas panels b, d and f are close up images at x 25,000 magnification.

SEM showed resting macrophages (in the absence of bacteria) as circular, uniform cells (Figure 1a/b(Fig. 1)) lacking pseudopodia. Following incubation with bacterial cells, the cellular morphology of macrophages substantially differed following treatment with *S. aureus* (Figure 1c/d(Fig. 1)) compared to *P. aeruginosa *(Figure 1e/f(Fig. 1)). After incubation of macrophages with *S. aureus* (Figure 1c/d(Fig. 1)), macrophages were not seen to produce the long linear pseudopodia consistently observed following incubation with *P. aeruginosa *(Figure 1e/f(Fig. 1)). The macrophages inoculated with *S. aureus* were instead much more globular and amorphous in shape.

**Figure 2:**
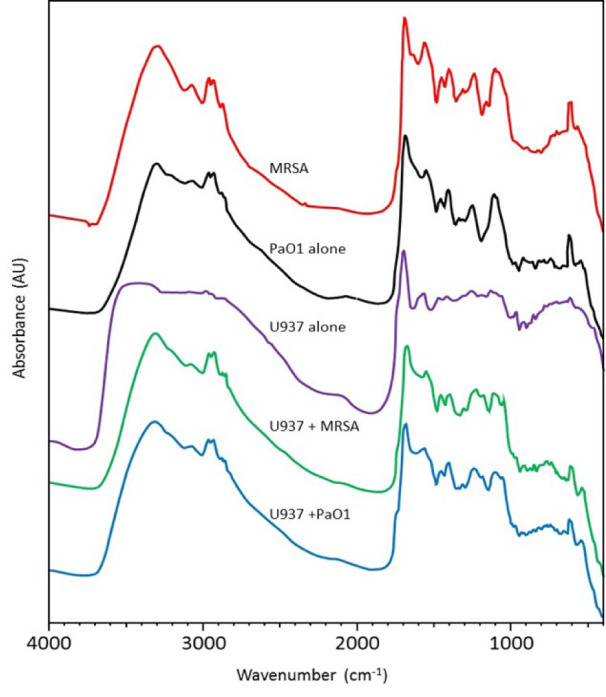
FTIR spectra of U937-derived macrophages alone, bacteria alone and macrophages incubated with bacteria. MRSA = methicillin-resistant *S. aureus*, Pa01 = *P. aeruginosa*

In order to determine why distinct surface changes in the macrophages had occurred following incubation with the different bacteria, FTIR was carried out on resting (control) macrophages in the absence of bacteria, bacteria alone and macrophages following incubation with bacteria. Analysis of the FTIR results demonstrated that both *S. aureus* and *P. aeruginosa* produced peaks at 3297 cm^-1 ^- 3295 cm^-1 ^(O-H stretch) and 1103 cm^-1^ - 1104 cm^-1^ (NH_2 _rocking). However, distinct bands were observed for *S. aureus* at 1556 cm^-1 ^(Amine II CN stretching) and 611 cm^-1^ (C-O vibrations Amide VI), and for* P. aeruginosa *at 914 cm^-1 ^(DNA, ribose vibration) (Figure 2(Fig. 2)).

The resting (control) macrophages demonstrated FTIR spectra at 3412 cm^-1 ^(O-H stretch), 1698 cm^-1 ^(C=O acidic stretch), 1566 cm^-1 ^(Amide II N-H bend, C-N bending) and 1130 cm^-1 ^(C-O-C stretching from CHOH). Following incubation of macrophages with *S. aureus*, there were differences in the spectra at 3309 cm^-1 ^(=CH), 2929 cm^-1^ (CH_2_ anti asymmetric stretch), 1227 cm^-1^ (PO_2_^-^ asymmetric stretch/ arachidonic acid) and 1118 cm^-1 ^(RNA ribose C-O stretching vibration). Following incubation with *P. aeruginosa, *there were distinct spectral peaks observed at 3510 cm^-1 ^(O-H stretch) and 1090 cm^-1 ^(C-O-H bend or PO_2 _symmetric stretch) (Table 2(Tab. 2); References in Table 2: Filip et al., 2009[10]; Matijašević et al., 2016[25]; Periathai and Rajagopal, 2014[32]; Ramalingam et al., 2016[35]; Salman et al., 2017[40]; Severcan et al., 2005[41]; Stuart, 2004[43]; Whitehead et al., 2011[47]).

**Figure 3:**
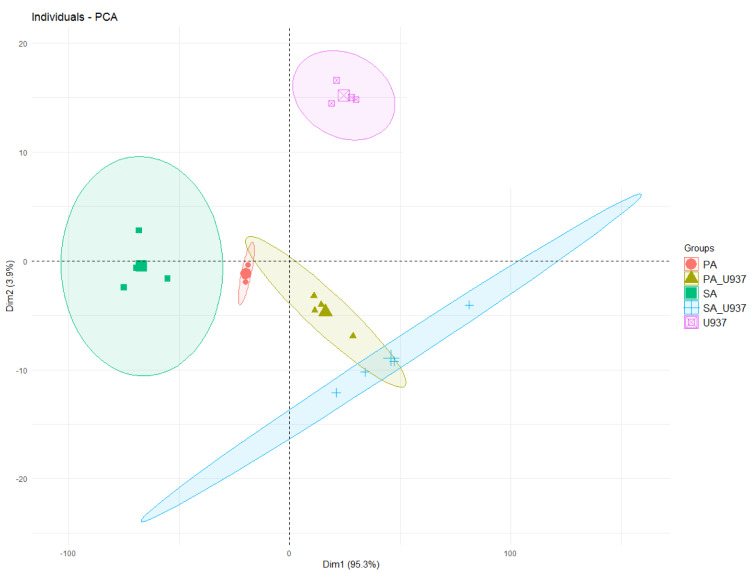
Scores' plot of the PCA (PC1 vs. PC2) of the FTIR of the samples. Ellipses represent a 95% confidence interval for the group. Points (samples) and ellipses are coloured: pink = U937 monocyte-derived macrophages only, red = *P. aeruginosa*, green = *S. aureus*, yellow-green = U937 monocyte-derived macrophages and *P. aeruginosa*, blue = U937 monocyte-derived macrophages and *S. aureus*).

The results from the FTIR were further analyzed using Principal Component Analysis (PCA). The PCA supported the distinct spectral findings and differing macrophage morphologies demonstrated by SEM, with >99 % of the variability in the FTIR spectra explained by the first two principal components (95.3 % and 3.9 % for principal component 1 and 2, respectively) (Figure 3(Fig. 3)).

## Discussion

Macrophages, as professional phagocytes of the immune system, engulf bacteria via phagocytosis, a process that involves membrane reorganization and remodeling events on the cell surface (Freeman and Grinstein, 2016[11]). In order to further understand the process of bacterial uptake by macrophages, the morphological and biochemical changes that occurred in macrophages, following interaction with two different bacterial genera, Gram-positive *S. aureus *and Gram-negative *P. aeruginosa *were investigated. Macrophages engulf bacteria through a series of events that involves dynamic reorganization and remodeling of the plasma membrane (Rubio et al., 2015[39]). To the authors' knowledge, the precise cellular response of macrophages to the microorganisms used in this study has not been fully elucidated to date (Rabehi et al., 2001[34]). However, it is known that the early response to pathogens may differ dramatically depending upon the inciting organism (Feezor et al., 2005[9]).

The results obtained from the initial host-pathogen assays demonstrated that the *S. aureus* recovery was significantly (P< 0.05) lower than *P. aeruginosa* suggesting that clearance of *S. aureus *was significantly greater than that of *P. aeruginosa*. This impairment in the uptake of *P. aeruginosa* was demonstrated by distinct morphological and biochemical changes in macrophages, observed under the SEM, following incubation with *P. aeruginosa*. Rabehi et al. (2001[34]) showed that activation of the inflammatory response by Gram-negative and Gram-positive bacteria can induce alternative pathways of intracellular signaling, which may contribute differences in the bacterial uptake efficiency and morphological/biochemical changes of macrophages observed in this study (Rabehi et al., 2001[34]). Gram-negative and Gram-positive bacteria have different cell wall biochemistries. *S. aureus *possesses a variety of secondary surface glycopolymers, including capsule polysaccharide, poly-β(1-6)-N acetylglucosamine, and teichoic acid (Weidenmaier and Lee, 2015[46]; Grunert et al., 2018[14]). In contrast, *P. aeruginosa *possesses bacterial LPS, with complex glycolipids providing characteristic components within the outer bacterial membrane (Heinrichs et al., 1998[17]).

The innate immune system has pattern recognition receptors (PRRs) which are able to sense different types of microbial pathogens, often through recognition of bacterial cell wall components, such as LPS from Gram-negative bacteria or lipoproteins from Gram-positive bacteria (Triantafilou and Triantafilou, 2002[45]; Nguyen and Götz, 2016[30]). Although phagocytosis is a receptor-mediated process, it also relies on spatial-temporal modification of lipids in the plasma membrane, such as phosphoinositides (PIs) and sphingolipids (Botelho et al., 2000[1]; Tafesse et al., 2015[44]). PRRs include the Toll-like receptor (TLR) and the C-type lectin families, which localize in lipid rafts and can be involved in membrane partitioning (Triantafilou and Triantafilou, 2002[45]). TLRs are expressed on immune cells and are able to distinguish bacterial cell wall components such as LPS from Gram-negative bacteria or lipoproteins from Gram-positive bacteria (Triantafilou and Triantafilou, 2002[45]). *S. aureus *and *P. aeruginosa *are recognized by distinct PRRs and may induce distinct spatial-temporal modification of lipids in host cell membranes. Such differences may lead to altered efficiency, morphology and biochemical profile of phagocytes. 

In the FTIR analysis, spectral peaks were observed in the regions that can be obscured by the presence of O-H molecules. One of the main difficulties of using mid-IR spectroscopy for biological applications is the presence of water in biological tissues and fluids. Water has a strong absorption over a broad range and it can mask the absorption of other tissue components (Minnes et al., 2017[26]). However, FTIR spectra at 3510 cm^-1^ and 3412 cm^-1^ may be indicative of the hydroxyl group of cell wall components such as carbohydrates, whilst the peak observed at 3309 cm^-1^ can relate to the degree of unsaturated phospholipid acyl chains.

From the FTIR spectra, *P. aeruginosa* demonstrated an individual peak that typically corresponded to DNA or RNA phosphate skeletal motions. *S. aureus *also demonstrated a small number of individual peaks suggesting the presence of Amine II and Amide VI molecular species. The resting macrophages demonstrated unique peaks indicative of the hydroxyl group of cell wall components, Amide I, Amide II and carbohydrates. 

Following the incubation of macrophages with *S. aureus*, there were new distinct peaks observed which demonstrated changes in the conformation of =CH, CH_2_ anti-asymmetric stretch, PO_2_^-^ asymmetric stretch / arachidonic acid and lipid molecules. These results suggest that changes occurred mainly in the phospholipid and lipid composition of the macrophage cell membranes. This may be due to a change in the degree of unsaturation in the phospholipid acyl chains, the fluidity of the macrophages and possibly to a change in the glycosidic linkages of the bacterial membrane (Mahoney et al., 1977[24]; Hagve, 1988[16]).

Incubation of the macrophages with *P. aeruginosa *determined distinct peaks in the O-H stretch, C-O-H bend and PO_2 _symmetric stretch. These results suggest that interaction of *P. aeruginosa* with the macrophages resulted in changes to the hydroxyl groups of cell wall components and carbohydrate / phospholipid composition of the macrophage membranes. The differences in biochemistry corresponding to these distinct peaks might indicate a tentative explanation for the more elongated, linear structure of the macrophage pseudopodia during *P. aeruginosa* engulfment. Given the lower activation of lipids observed in the macrophage membrane and the higher number of *P. aeruginosa *recovered from the macrophages, this may suggest that *P. aeruginosa* can in part prevent macrophages from engaging with the lipid membrane rafts required for phagocytosis. However, this hypothesis clearly needs further investigations.

Following PCA analysis, there were clear and distinct sample groupings into bacteria alone, resting macrophages (in the absence of bacteria) and macrophages interacting with bacteria. The principal component two (PC2) and peaks in the FTIR spectra were the main differentiators between resting macrophages and macrophages interacting with bacteria. Furthermore, *P. aeruginosa* and *S. aureus* spanned distinct areas in the scores plot. Differences in FTIR peaks were observed for both *P. aeruginosa* and *S. aureus *following interaction with macrophages. Similar to incubation of macrophages with *P. aeruginosa* in this study, mammalian cells of acute myelogenous leukemia demonstrated a peak at 1240 cm^-1^ that corresponded to symmetric and asymmetric PO_2_ stretching vibration of phosphodiester linkages of the polynucleotide chains (Liu et al., 2003[23]). In experiments where lymphocytes were stimulated with a mitogen (PHA), the band associated with lymphocyte activation was shown to be the *v_as_* PO^-2^ mode at 1244 cm^-1^ that is assigned to the phosphodiester backbone. Moreover, the *v_s_*PO^-2 ^at 1080 cm^-1^ (1090 cm^-1^ in our studies) has not only been associated to the phosphodiester backbone, but is also indicative of leukocyte activation (Wood et al., 2000[48]).

Changes in membrane phospholipid fatty acid composition might be expected to influence alterations in the physical properties of the macrophage membrane, such as membrane order (“fluidity”) and raft structure due to the relatively high amount of arachidonic acid in immune cell membrane phospholipids (Calder, 2008[2]). Changing the fatty acid composition of immune cells affects phagocytosis, T cell signaling and antigen presentation capability, and these effects appear to be mediated at the membrane level, suggesting important roles of fatty acids in membrane order, lipid raft structure and function, and membrane trafficking (Calder, 2008[2]). With regards to macrophages, it is known that the spatio-temporal orchestration of localized phosphoinositide generation is crucial for phagocytosis (Kannan, 2009[19]). *In vitro* studies have demonstrated that alterations in phagocyte membrane fatty acid composition are associated with altered phagocytic capacity of immune cells seen during the uptake of both *P. aeruginosa *and *S. aureus* (Calder, 2008[2]). Furthermore, *in vitro* studies carried out using murine macrophages have shown that enrichment of cell membranes with unsaturated fatty acids increases the phagocytic capacity of those cells (Calder et al., 1990[3]). Given the fatty acid profile of phospholipids influences membrane flexibility, the peak in unsaturated PL acyl chains in the FTIR spectra of macrophages incubated with *S. aureus* is in concordance with the significantly increased uptake of *S. aureus* in this study compared to *P. aeruginosa*. Arachidonic acid is a ubiquitous component of every mammalian cell. This polyunsaturated fatty acid serves important biochemical roles as a carbon fatty acid with four double bonds (Chaplin, 2011[4]). The ν(C=O) stretching vibration may indicate the dimer form of arachidonic acid (Gocen et al., 2018[13]), and this peak, which was observed following macrophage interaction with *S. aureus*, may be indicative of changes in the arachidonic acid in the macrophage lipid membrane.

A number of phagocytic receptors have been localized or are recruited upon activation to membrane microdomains called lipid rafts. Lipid rafts have been implicated in numerous cellular processes, and have been reported to be critical for pathogen internalization into cells (Lafont and Van Der Goot, 2005[22]). Kannan (2009[19]) demonstrated that signals for phagocytosis were initiated by active reorganization of lipid rafts. Currently, lipid rafts are thought to allow different protein-lipid and protein-protein interactions that temporarily compartmentalize the plasma membrane. Lipid rafts are characterized by a high cholesterol and glycosphingolipid content. In general, lipid rafts, caveolae and ceramide-enriched membrane domains are characterized by a reduced lateral mobility, a change of the membrane thickness and, in particular, a composition different from other parts of the membrane, the latter feature of which seems to be of particular importance to permit these domains to function as a sorting domain for proteins (Riethmüller et al., 2006[36]). Whether *S. aureus* and *P. aeruginosa* have distinct interactions with lipid rafts to alter efficiency, morphology or biochemical profile of phagocytes warrants further investigation. Indeed, *P. aeruginosa* appears to signal through lipid rafts to down-regulate the host immune response (Lafont et al., 2004[21]), but further work is needed to confirm whether this mechanism contributed to the impaired engulfment of *P. aeruginosa* in this study.

## Conclusions

To conclude, the surface morphology and biochemistry of macrophages were different following incubation with *S. aureus* compared to *P. aeruginosa *with lower survival of *S. aureus* compared to *P. aeruginosa* following a 3-hour host-pathogen interaction*. *After incubation with *S. aureus*, macrophages were more globular and amorphous whereas long linear pseudopodia were observed following incubation with *P. aeruginosa. *Distinct FTIR spectra, mainly relating to lipids and hydroxyl groups of cell membranes were identified in macrophages following uptake of *P. aeruginosa*. In contrast, distinct phospholipid and lipid FTIR spectra were detected in macrophages following engulfment of *S. aureus*. These findings suggest that bacteria of different genera invoke diverse biochemical changes in the macrophages following engulfment. However, whether the distinct biochemical components of the bacterial cell wall (Gram-negative versus Gram-positive) influenced the morphology and hence the phagocytic ability of macrophages in a pathogen-specific manner warrants further investigation.

## Funding

This work was funded by Manchester Metropolitan University.

## Conflict of interest

The authors declare that there is no conflict of interest.

## Authors’ contributions

MEM carried out the experimental work and CL was involved in the FTIR analysis. LP carried out the PCA. ND-H and AB contributed to the writing of the paper. KW and JA designed the study concept, managed the team and oversaw the writing of the paper.

## Figures and Tables

**Table 1:**
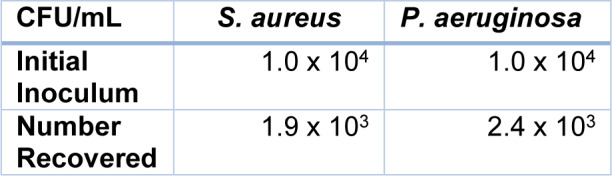
Recovery (CFU/ml) of bacteria (*P. aeruginosa*, *S. aureus*) following incubation with U937-derived macrophages for 3 hours

**Table 2:**
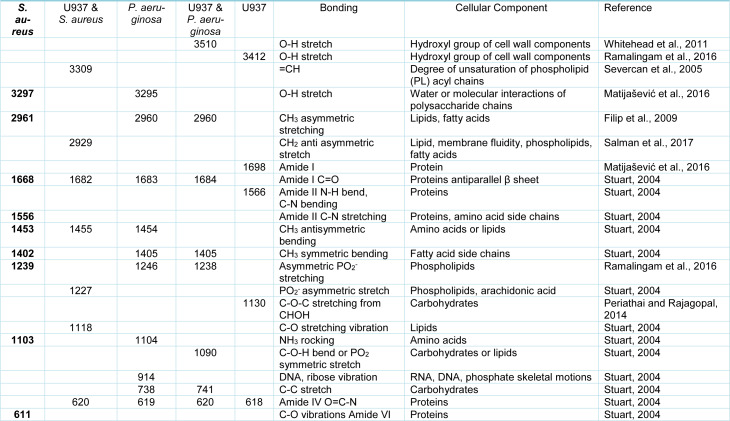
Changes in the FTIR spectra of the macrophages following incubation with bacteria
